# The Spectrum of Extra-intestinal Manifestation of Crohn's Disease

**DOI:** 10.7759/cureus.6928

**Published:** 2020-02-10

**Authors:** Laraib Jumani, Deepak Kataria, Muhammad Umer Ahmed, Mir Ali Asghar Shah, Kunal Raja, Faizan Shaukat

**Affiliations:** 1 Internal Medicine, Pakistan Institute of Medical Sciences, Islamabad, PAK; 2 Internal Medicine, Shaheed Mohtarma Benazir Bhutto Medical University, Larkana, PAK; 3 Internal Medicine, Ziauddin University, Karachi, PAK; 4 Internal Medicine, Jinnah Postgraduate Medical Center, Karachi, PAK; 5 Internal Medicine, Jinnah Post Graduate Medical Center, Karachi, PAK

**Keywords:** crohn's disease, inflammatory bowel disease, extraintestinal manifestations

## Abstract

Introduction

Extra-intestinal manifestations (EIM) play an important role in the mortality, morbidity, and quality of life in patients with Crohn’s disease (CD). Understanding the prevalence and clinical course of these manifestations is important to understand and manage CD.

Materials and methods

The hospital records of 103 patients diagnosed with CD between July 2016 and December 2019 at a tertiary care hospital in Pakistan were reviewed retrospectively. Baseline demographic and clinical characteristics including sex, age, follow-up duration, CD phenotype at diagnosis, clinical features, and course of EIMs were noted. The diagnosis of CD was based on clinical features in combination with endoscopic and radiologic findings. CD phenotype at diagnosis was assessed using the Montreal Classification by the World Congress of Gastroenterology (WCOG).

Results

The mean age at diagnosis of CD was 31 ±8 years. The most common age group as per the Montreal classification at diagnosis was 17-40 years (68.93%). The most common localization of disease was ileocolonic (70.87%), and the most common disease behavior was non-stricturing and non-penetrating (82.52%). In this study, 41 (39.8%) patients had a minimum of one EIM. The most common EIM was anal skin tags (29.12%), while 24 (23.30%) patients had elevated liver function tests (LFTs), three (2.91%) had peripheral arthritis, and 12 (11.65%) had cutaneous manifestations, the most common being erythema nodosum (7.76%). The most common ophthalmological manifestation was anterior uveitis (3.88%).

Conclusions

EIMs are prevalent in CD patients in Pakistan, yet very little is known about them. Further large-scale studies are needed to assess the frequency and impact of EIMs on patients with CD.

## Introduction

Crohn’s disease (CD) is a chronic, relapsing inflammatory bowel disease (IBD), which is characterized by granulomatous transmural inflammation. It might affect any part of the gastrointestinal tract, but most commonly involves the ileum, colon, or ileocolonic region [1]. The Montreal Classification by the World Congress of Gastroenterology (WCOG) categorizes CD based on factors including age at diagnosis, disease location, and behavior [2]. CD symptoms are non-specific and vary widely as presentation includes general symptoms such as fever, fatigue, and weight loss, accompanied by intestinal symptoms such as abdominal pain and chronic diarrhea due to inflammation of intestinal mucosa [3,4]. Along with intestinal symptoms, CD also involves extra-intestinal manifestations (EIMs) in 6-36% of patients. The most commonly affected organs are the joints, skin, uvea, blood, and hepatobiliary system. The most common form of EIM is arthropathy. The most diverse EIMs are cutaneous manifestations, which may include characteristic findings such as erythema nodosum and pyoderma gangrenosum as well as other non-specific signs such as cellulitis and lichen planus [5]. EIMs contribute significantly to morbidity and mortality in CD [5]. Very little data are available regarding EIMs in IBD in Pakistan. Understanding the prevalence and clinical course of the EIMs in patients with CD will significantly assist physicians in the management of CD.

## Materials and methods

The hospital records of 103 patients diagnosed with CD between July 2016 and December 2019 at a tertiary care hospital in Pakistan were reviewed retrospectively. The following demographic and clinical characteristics were included: sex, age, follow-up duration, CD phenotype at diagnosis, clinical features, and frequency of EMIs. The diagnosis of CD was based on clinical features in combination with endoscopic and radiologic findings. CD phenotype at diagnosis was assessed using the Montreal Classification (Table 1) [2].

**Table 1:** The Montreal Classification of inflammatory bowel disease A1, A2, A3: different categories based on age at diagnosis; L1, L2, L3: different categories based on disease location; B1, B2, B3: different categories based on disease behavior *P is added to modify the B1-B3 classification when concomitant perianal disease is present

The Montreal Classification		
Age at diagnosis, years	A1	<17
	A2	17-40
	A3	>40
Disease location	L1	Terminal ileum
	L2	Colon
	L3	Ileocolonic
	L4	Isolated upper gastrointestinal
Behavior	B1	Non-structuring, non-penetrating
	B2	Structuring
	B3	Penetrating
	P	Perianal disease modifier*

Pain, swelling, redness, and tenderness of one or more joints were identified as arthritis. Erythema nodosum was diagnosed when tender, red nodules were present, mainly on the extensor surface of the legs. Skin and eye lesions were identified with the help of dermatologists and ophthalmologists.

## Results

In our study, 103 complete records of patients were identified and included; 53 (51.45%) patients were male, and 50(48.55%) were female. The mean age at diagnosis of CD was 31 ±8 years. The most common age group as per the Montreal Classification was 17-40 years at diagnosis (68.93%). The most common localization of disease was the ileocolonic region (70.87%), and the most common disease behavior was non-stricturing, non-penetrating (82.52%) (Table 2).

**Table 2:** Patient characteristics in the study based on Montreal Classification A1, A2, A3: different categories based on age at diagnosis; L1, L2, L3: different categories based on disease location; B1, B2, B3: different categories based on disease behavior *P is added to modify the B1-B3 classification when concomitant perianal disease is present

Patient details as per Montreal Classification			Number of patients (%)
Age at diagnosis, years	A1	<17	23 (22.33%)
	A2	17-40	71 (68.93%)
	A3	>40	9 (8.73%)
Disease Location	L1	Terminal ileum	14 (13.59%)
	L2	Colon	16 (15.53%)
	L3	Ileocolonic	73 (70.87%)
	L4	Isolated upper gastrointestinal	0
Behavior	B1	Non-structuring, non-penetrating	85 (82.52%)
	B2	Structuring	12 (11.65%)
	B3	Penetrating	6 (5.82%)
	P	Perianal disease modifier*	48 (46.6%)

The most common EIM observed was anal skin tags (29.12%). Twenty-four (23.30%) patients had elevated liver function tests, while three had peripheral arthritis, and 13 (12.62%) had cutaneous manifestations, the most common being erythema nodosum (7.76%). The most common ophthalmologic manifestation was anterior uveitis (3.88%) (Table 3).

**Table 3:** Frequency of extra-intestinal manifestations in study participants LFT: liver function test

Extra-intestinal manifestations	Number of patients (%)
Anal skin tags	30 (29.12%)
Elevated LFTs	24 (23.30%)
Peripheral arthritis	13 (12.62%)
Erythema nodosum	08 (7.76%)
Pyoderma gangrenous	03 (2.91 %)
Psoriasis	01 (0.97%)
Ezcema	01 (0.97%)
Anterior uveitis	04 (3.88%)
Scleritis	03 (2.91%)
Conjunctivitis	03 (2.91%)

## Discussion

In our study, the mean age at diagnosis was 31 ±8 years, with the most common age group being 17-40 years. This is consistent with various studies that state that CD is usually diagnosed in the second or third decade of life [6]. In this study, the disease was most commonly localized in the ileocolonic region (70.87%). It is important to identify the site of disease as it is associated with complications and future need for surgery. Disease localized in the ileum is more commonly associated with stricturing and fistulizing CD phenotypes [7]. Along with a higher rate of complications, disease localized in the ileum is associated with the need for CD-related surgery including small bowel resection, strictureplasty, and abscess drainage [7]. In this study, 41 (39.8%) patients had a minimum of one EIM. This was comparable to findings from a study conducted in India, which stated a prevalence of 38% EIM in IBD [8]. The prevalence in our study was slightly higher. Lakatos et al. stated that 20-25% of patients with CD had one major EIM [5]. Despite their prevalence, the pathogenesis of EIMs in patients with CD is not completely understood. One of the more accepted theories is that patients’ gastrointestinal mucosa can trigger an immune response in extraintestinal sites due to common epitopes in genetically susceptible individuals [9-11].

The most common EIM found in our study was anal skin tags; however, their association with disease activity is still controversial. While there have been reports of anal skin tags being more prominent when CD is active, other studies have disputed this finding [12]. Anal skin tags are painless and can vary from being narrow to broad; they are usually present in multiple numbers. However, they are usually benign and require no intervention [13].

Overall, cutaneous manifestations were reported in 13 patients (12.62%). The most common cutaneous manifestation was erythema nodosum (7.76%). The pathogenesis of erythema nodosum as an EIM of CD can be explained as an abnormal immune response that causes a release of cytokines including tumor necrosis factors and interleukins. These inflammatory signals are directed against common epitopes between intestinal bacteria and skin [8]. The nodules’ appearance correlates with intestinal activity; they rarely precede the onset of CD or appear during quiescent phases of the disease. Cutaneous lesions disappear with the treatment of CD [14].

Peripheral arthritis was reported in 13 participants (12.62%), which is low in comparison to a regional study conducted in India, which reported a prevalence of 23% [8]. Peripheral arthropathies occur in 5-20% of patients with IBD and are generally most common in CD [15]. The role of various genetic factors such as class I and II molecules of major histocompatibility complex (HLA) in the pathogenesis of IBD including joint involvement has been studied. In patients with IBD, some HLA alleles are associated with increased risk for the development of certain EIMs [16].

Ocular manifestations were found in 10 (9.70%) patients, compared to 13% in a study by Bandyopadhyay et al. [8]. Ocular EIMs are mostly inflammatory in origin and more prevalent in patients with colonic or ileocolonic involvement [8]. The main ocular manifestations include episcleritis, scleritis, and anterior uveitis, all three of which were found in our study [17].

This is the first study conducted in Pakistan that examines EIMs in CD. Despite the study’s small sample size, the most common EIMs were all observed in our cohort. However, the study has its limitations. Firstly, since it was a single-institution study, results should be extrapolated to the general population with caution. Secondly, the retrospective nature of the study meant that the relationship between EIM and disease activity could not be inferred.

The prevalence of EIMs was higher in Pakistan compared to instances found in the literature that examined its prevalence in the west. EIMs can involve many vital organs and systems. The pathogenesis of EIMs, although not fully understood, is related to the complex interaction between an adaptive immune response directed against intestinal and extraintestinal sites as they share common epitopes.

## Conclusions

It is estimated that one-third of patients with CD exhibit EIMs. It is important to involve specialists such as dermatologists and ophthalmologists early in the management of organs involved. It is also important to counsel patients about EIMs and their relationship to disease activity. Further large-scale studies are warranted to better understand EIMs in CD.
